# Sex-divergent trends in cardiorespiratory endurance after the COVID-19 pandemic: a 21-year interrupted time series analysis of Chinese university students

**DOI:** 10.3389/fpubh.2026.1910319

**Published:** 2026-10-05

**Authors:** Chao Song, Xinkui Hu, Ningyan Lu, Wei Gao, Feng Xie, Yanqiang Yin, Linlin Yan, Duming Wang, Zhe Wang, Guomin Li

**Affiliations:** 1 Department of Psychology, Zhejiang Sci-Tech University, Hangzhou, Zhejiang, China; 2 Department of Physical Education, Zhejiang Sci-Tech University, Hangzhou, Zhejiang, China

**Keywords:** cardiorespiratory endurance, China, COVID-19, interrupted time series, physical fitness, population trends, sex differences, university students

## Abstract

**Introduction:**

Studies of COVID-19 and physical fitness in Chinese university students rarely extend beyond 2022, and none has formally tested whether post-pandemic trends differ by sex.

**Methods:**

Using 21 years of routinely collected fitness data (2005–2025), we examined indicator-specific change and sex differences in population-level trends in a repeated cross-sectional design. The cardiorespiratory analytic sample comprised 297,565 valid records from 91,686 full-time undergraduates at one comprehensive university in Zhejiang Province, China. Six indicators were classified by data availability and suitability for interrupted time series (ITS) analysis; cardiorespiratory endurance (1000-m run for men, 800-m for women) was pre-specified as the primary outcome and body weight was analysed within the same framework as a negative control. The unit of analysis was the annual grade-standardised mean, giving 18 time points per sex. Single-breakpoint models estimated the immediate level change at 2020 (*β*_2_) and the change in annual trend thereafter (*β*_3_); a sex × trend interaction tested whether post-2020 trends differed between women and men; Benjamini–Hochberg correction was applied across five confirmatory tests.

**Results:**

Run times in 2020 rose above counterfactual projections in both sexes (women +9.60 s, 95% CI 2.58–16.62, *p* = 0.011, *q* = 0.019; men +13.02 s, 95% CI 4.76–21.28, *p* = 0.005, *q* = 0.019), although this immediate change was attenuated and no longer significant when fourth-year students, tested in May 2020 rather than December, were excluded. Post-2020 trends differed by sex (interaction *p* = 0.030, *q* = 0.037) and this difference was robust to that exclusion. In men the post-2020 trend did not differ significantly from the pre-pandemic trend (*β*_3_ = −0.47 s/year, *p* = 0.609), with the 2025 annual mean remaining about 14.9 s above the counterfactual projection. In women the trend was significantly steeper (*β*_3_ = +2.25 s/year, *p* = 0.011, *q* = 0.019), widening the gap to about 26.0 s by 2025. Body weight rose steadily across the whole period but showed no change attributable to the pandemic (level change *p* = 0.31 and 0.70; trend change *p* = 0.57 and 0.92 in women and men), which is consistent with the cardiorespiratory result reflecting something other than an artefact of the model.

**Discussion:**

Cardiorespiratory endurance was the only indicator analysed by ITS that showed a sustained pandemic-associated change, and its subsequent trend diverged by sex. These are aggregate population trends rather than individual trajectories, but they suggest that post-pandemic aerobic programming may need to be sex-specific, and that continuity of aerobic activity deserves explicit attention in emergency preparedness planning.

## 1 Introduction

The COVID-19 pandemic and the measures used to contain it disrupted physical activity among adolescents and young adults worldwide (1, 2). China’s response was distinctive in both its duration and its intensity, and understanding what it did to young people’s fitness requires some familiarity with what the policy actually involved.

From early 2020 until December 2022, China pursued a “dynamic zero-COVID” policy whose objective was to eliminate transmission chains rather than to slow their spread. On university campuses this meant controlled entry and, during outbreak periods, closure to visitors; suspension of in-person physical education classes and of organised and competitive sport; closure of indoor sports facilities and restricted access to outdoor tracks and fields; periodic transitions to online instruction; and mass testing with localised quarantine of buildings or campuses when cases were detected (3, 4). Restrictions were applied dynamically in response to local case detection, so their intensity varied over time and between cities rather than following a fixed national schedule. The policy was then lifted abruptly in December 2022, which produced an unusually sharp transition back to normal campus activity and a comparatively well-defined reopening period for study.

A growing body of evidence has documented declines in cardiorespiratory endurance among Chinese university students during the pandemic, with reported increases in 800-m and 1,000-m run times of 8–17% across multiple regions (5–10). Several studies confirmed that these impairments persisted into 2022 (8, 11). Yet despite this rapid accumulation of evidence, three substantive questions remain unanswered.

First, the overwhelming majority of published studies conclude in 2022 or earlier—before China’s December 2022 reopening—and therefore cannot describe what happened afterwards (5–12). The 2023–2025 window, during which campus life and physical activity in China returned to relative normalcy, has not been formally analyzed at scale. Whether the fitness deficits incurred during the pandemic resolved, persisted, or worsened after restrictions were lifted remains uncharacterized at the population level.

Second, most existing studies examine only one or two indicators in isolation (5, 6, 8, 9), leaving unresolved which components of physical fitness are most vulnerable to acute activity restriction and which are robust. Aerobic capacity is known to deteriorate rapidly with detraining (13, 14), whereas other components, such as muscular strength and body composition, are thought to be more resilient over short interruptions (15). Whether this differential vulnerability is empirically detectable in a large student population—and whether it would justify component-specific rather than uniform intervention strategies—has not been systematically tested within a single analytical framework.

Third, although sex differences are documented both in physiological responses to detraining (16, 17) and in the psychosocial burden of the pandemic (18–20), we are not aware of any study that has formally tested whether population-level fitness trends after the lifting of restrictions differ between women and men. This matters practically, because post-pandemic health programmes have generally been designed as universal interventions and the evidence needed to judge whether sex-specific approaches are warranted does not currently exist.

The present study addresses these three questions using 21 years of routinely collected, methodologically consistent physical fitness data from a single comprehensive university in Zhejiang Province, China (2005–2025). The single-site design—often regarded as a limitation—is here a deliberate methodological strength: testing was conducted by one physical education team using equipment and protocols that have remained materially unchanged across two decades, providing a level of measurement consistency rarely attainable in multi-site studies and essential for distinguishing pandemic-specific effects from secular trends. The extended pre-pandemic baseline (2008–2019 for the primary outcome) enables formal counterfactual modeling rather than simple before-and-after comparison, and the large analytic sample (108,259 students, 350,976 measurements) confers the statistical power required to test for sex differences in post-2020 trends directly.

Our aims were to estimate the immediate change in cardiorespiratory endurance at 2020 and the change in annual trend thereafter; to establish whether that pattern was specific to cardiorespiratory endurance or common to other fitness components, using body weight analysed in the same framework as a negative control; and to test formally whether post-2020 trends differed between women and men. Participant flow is shown in Figure 1.

**Figure 1:**
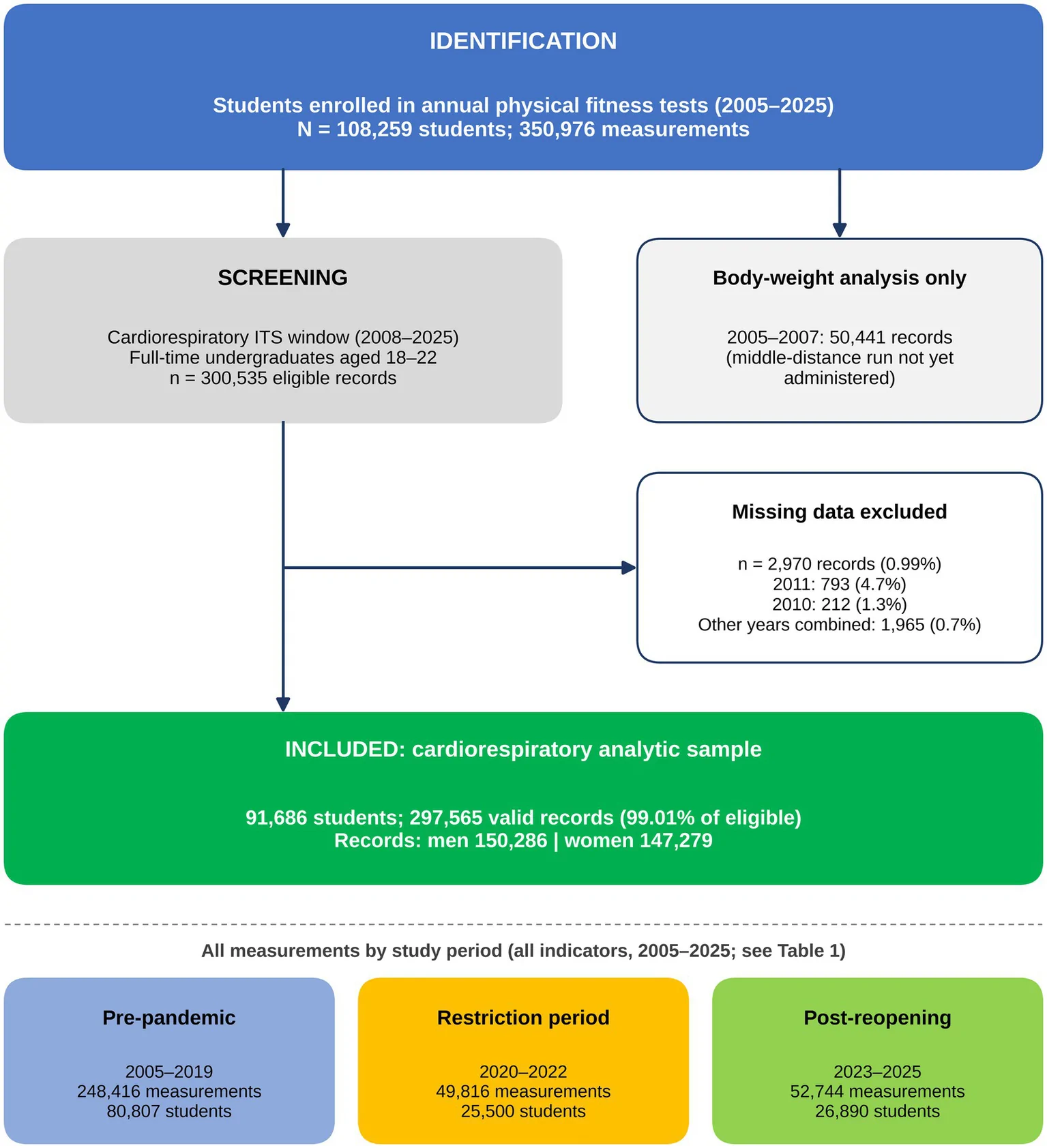
Participant flow and analytical sample derivation (2005–2025). Identification, eligibility screening and exclusions (2,970 records; 0.99%) for the cardiorespiratory analysis, the final cardiorespiratory analytic sample (297,565 valid records from 91,686 students), and the 50,441 records from 2005–2007 retained for the body-weight analysis only. The lower boxes show all measurements (all indicators) by study period, as in Table 1.

## 2 Materials and methods

### 2.1 Study design and ethics

We conducted a repeated cross-sectional study with interrupted time series (ITS) analysis of annual physical fitness test data collected from 2005 to 2025 at a comprehensive public university in Zhejiang Province, China. Testing followed standard institutional practice: first-through third-year students completed the annual assessment each December, whereas fourth-year students were tested at the end of the spring semester (May). Reporting follows the STROBE guideline for cross-sectional studies (21); a completed checklist is provided as a Supplementary file. As a retrospective analysis of routinely collected, fully de-identified educational records, the study was approved by the Research Ethics Committee of the Department of Psychology, Zhejiang Sci-Tech University (approval number 202501H001), with a waiver of informed consent. The data derive from the annual student physical fitness testing mandated by the Chinese Ministry of Education’s National Student Physical Fitness Standard (22), which the university has administered continuously since 2005.

Three periods were defined in advance: a pre-pandemic baseline (2005–February 2020); the restriction period (February 2020–February 2023), corresponding to the dynamic zero-COVID policy; and the post-reopening period (March 2023–2025).

### 2.2 Study setting and sample

The participating institution is a comprehensive public university with stable annual enrolment of approximately 28,000 undergraduates. Testing was performed by a single physical education teaching team using standardised equipment that has remained materially unchanged across the 21-year window.

Records were eligible for inclusion if the student was enrolled as a full-time undergraduate in any academic year from 2005 to 2025, was aged 18–22 years, participated in the mandatory annual fitness test, and had a valid and parseable value for the indicator under analysis. Records were excluded if the relevant value was missing, zero, or unparseable; if the record carried a documented medical exemption or an explicit non-participation entry (“not tested,” “abroad,” “exchange student”); or, for the cardiorespiratory analysis, if the record fell outside 2008–2025, before which the middle-distance run was not administered consistently.

No *a priori* sample size calculation was performed. The study analyses a complete administrative census: every student tested in every year was included and no sampling took place. Across the full window, 108,259 unique students contributed 350,976 measurements; for the cardiorespiratory ITS analysis, 297,565 valid records from 91,686 students were retained. As set out in section 2.4, the constraint on statistical power in the ITS models is the number of annual time points rather than the number of individual records. Sample characteristics by period are given in Table 1 and participant flow in Figure 1.

**Table 1:** Sample characteristics by study period (2005–2025).

Study period	Measurements	Unique students	Male, *n* (%)	Female, *n* (%)	Age, years
Pre-pandemic (2005–2019)	248,416	80,807	128,336 (51.7)	120,080 (48.3)	18–22
Restriction period (2020–2022)	49,816	25,500	25,069 (50.3)	24,747 (49.7)	18–22
Post-reopening (2023–2025)	52,744	26,890	27,773 (52.7)	24,971 (47.3)	18–22
Total	350,976	108,259	181,178 (51.6)	169,798 (48.4)	18–22

Period-specific unique-student counts sum to more than the overall total because the four-year undergraduate structure means many students contributed measurements in more than one period (mean 3.24 measurements per unique student across the 21-year window). By unique student (sex assigned as the most frequently recorded value), the full sample comprised 56,177 male (51.9%) and 52,082 female (48.1%) students.

### 2.3 Physical fitness assessments and indicator tiering

Six indicators were extracted from the annual test battery, each assessing a distinct component of health-related fitness or motor ability (23): cardiorespiratory endurance (1000-m run for men, 800-m for women); body composition (body weight, kg); lower-limb explosive power (standing long jump, cm); speed (50-m sprint, s); flexibility (sit-and-reach, cm); and muscular endurance, assessed with a sex-specific test (pull-ups for men, one-minute sit-ups for women).

Testing followed the protocols specified in the National Student Physical Fitness Standard (22), a battery whose reliability in Chinese college populations has been documented previously (24, 25). All students completed the battery in a fixed order on a single testing day, with the sequence designed so that measurements least affected by fatigue were taken first and the most demanding item last. Anthropometric measurements (height and body weight) were therefore performed before any physical performance test, followed by the sit-and-reach, the 50-m sprint, the standing long jump, the muscular endurance test, and finally the middle-distance run. A supervised standardised warm-up of approximately 10 min preceded testing, and a minimum 10-min rest was enforced before the middle-distance run. Testing took place on a 400-m outdoor synthetic track between 08:00 and 17:00 and was postponed in rain, extreme heat, or other adverse conditions. First-through third-year students were tested each December and fourth-year students at the end of the spring semester in May; this schedule was fixed and identical in every study year.

The muscular endurance indicator warrants explicit comment. Pull-ups and sit-ups are not equivalent tests and are not physiologically comparable; the pairing is prescribed by the national standard and cannot be altered retrospectively. Accordingly, no comparison of this indicator was made between sexes at any point, and all inference is restricted to change within each sex relative to that sex’s own pre-pandemic baseline.

The National Student Physical Fitness Standard also defines age- and sex-specific scoring tables for the middle-distance run (22), with separate thresholds for first- and second-year and for third- and fourth-year students. For women, the 60-point (pass) threshold is 4 min 34 s in years 1–2 and 4 min 32 s in years 3–4, and the 80-point (good) threshold is 3 min 44 s and 3 min 42 s respectively; for men, the corresponding 1,000-m thresholds are 4 min 32 s and 4 min 30 s (pass) and 3 min 42 s and 3 min 40 s (good). To obtain a single institution-level threshold comparable with our grade-standardised series, we combined the two grade bands using the same fixed weights applied in the standardisation (years 1–2 = 0.5134, years 3–4 = 0.4866), giving a pass threshold of 273 s for the 800-m run and 271 s for the 1,000-m run and a good threshold of 223 s and 221 s, respectively. Because the 50-s interval between these thresholds spans 20 scoring points in both sexes, 2.5 s of running time corresponds to one point on the national scale. We use this correspondence in the Results to express the observed changes in units that carry administrative meaning; it is a linear approximation within the pass-to-good band and is not extrapolated beyond it.

Indicators were classified into three tiers according to data availability and the suitability of ITS analysis (Supplementary Figure S1). Tier 1 (body weight) had 21-year continuous data (2005–2025), passed standard ITS diagnostic criteria, and served as an in-framework negative control: because body weight follows long-term secular dynamics, a pandemic-specific ITS effect (an abrupt level or slope change at 2020) would not be expected, and its absence provides evidence that any effect detected for the primary outcome is not a methodological artifact. Tier 2 (cardiorespiratory endurance) had 18-year continuous data (2008–2025), passed ITS diagnostics, and was designated *a priori* as the primary outcome given its established sensitivity to detraining (13, 14). Tier 3 comprised four indicators (standing long jump from 2005; 50-m sprint, sit-and-reach, and muscular endurance from 2013) for which ITS assumptions were violated or fewer than 12 pre-pandemic data points were available; these were assessed by pre-pandemic baseline comparison rather than ITS modeling. This tiering was imposed by the data rather than chosen, and it means that the comparison across indicators is not methodologically uniform, a limitation we return to in the Discussion.

### 2.4 Statistical analysis

#### 2.4.1 Grade standardization

The unit of analysis in all ITS models is the annual grade-standardised mean, giving 18 annual time points per sex for cardiorespiratory endurance (12 pre-pandemic, three during restrictions, three after reopening) and 21 for body weight. The large individual-level sample determines the precision with which each annual mean is estimated; it does not increase the number of observations available to the time series models. Statistical power for the ITS parameters is therefore governed by the number of annual points.

Because the grade composition of the tested population varied across years—owing to enrollment fluctuations and, during the pandemic, scheduling adjustments—all annual means were grade-standardized before analysis using fixed reference weights derived from the long-term enrollment distribution: Year 1 = 0.2585, Year 2 = 0.2548, Year 3 = 0.2443, Year 4 = 0.2423. This procedure controls for confounding from changes in grade distribution while preserving the within-grade temporal signal.

#### 2.4.2 Primary analysis: single-breakpoint ITS

The primary model was a single-breakpoint interrupted time series specified as:


$$Y_{t}=\beta_{0}+\beta_{1}\times {\text{Time}}_{t}+\beta_{2}\times {\text{Intervention}}_{t}+\beta_{3}\times \text{Time}\_{\text{after}}_{t}+\varepsilon_{t}$$

where *Y_t_* is the grade-standardized annual mean of the indicator, Time*_t_* is the calendar year centered at the series origin, Intervention*_t_* is a binary pandemic indicator (0 before 2020, 1 from 2020 onward), and Time_after*_t_* counts years from the 2020 breakpoint. *β*_1_ estimates the pre-pandemic annual trend, *β*_2_ the immediate level change at 2020, and *β*_3_ the change in the annual trend after 2020, expressed as a deviation from the projected pre-pandemic trajectory (26, 27). Models were estimated by ordinary least squares (OLS). Newey–West heteroskedasticity- and autocorrelation-consistent standard errors (lag 2) were computed as a sensitivity check for residual autocorrelation (28); inference was based on OLS estimates, and the significance of all primary inferences was unchanged under the robust specification. We emphasise that *β*_3_ should not be read as a “recovery” parameter: the window it spans (2020–2025) contains both the restriction period and the post-reopening period, and only three annual observations fall after full reopening.

Two specification checks were carried out in response to peer review. First, a model with a second breakpoint at 2023, corresponding to the lifting of restrictions, was compared with the single-breakpoint model using AIC, BIC, and nested *F*-tests; the single-breakpoint model was retained in both sexes (Supplementary Tables S1, S6). Second, because annual means are estimated with differing precision in principle, all primary models were refitted with each annual mean weighted by its underlying sample size; estimates were essentially unchanged (Supplementary Table S7).

#### 2.4.3 Secondary analyses

Indicator comparison. ITS models were fitted to each Tier 1 and Tier 2 indicator. Tier 3 indicators were assessed by pre-pandemic baseline comparison (2017–2019 reference) and summarized with Cohen’s *d* using pooled individual-level standard deviations (29).

Sex differences in post-2020 trends. Sex-specific ITS models were fitted, and a pooled model incorporating a sex × time-after interaction term formally tested the null hypothesis of equal post-2020 trends (30).

Multiplicity. The five confirmatory cardiorespiratory tests (immediate and slope-change effects in each sex, plus the sex × slope interaction) were corrected for multiple comparisons using the Benjamini–Hochberg false discovery rate procedure (31); body-weight negative-control tests were not included in this confirmatory family. The unadjusted and FDR-adjusted *p*-values for all five tests are reported in Supplementary Table S4.

#### 2.4.4 Sensitivity and robustness checks

Five checks were performed: alternative pre-pandemic baseline windows (2015–2019, 2016–2019, 2017–2019; Supplementary Table S2); grade-stratified models with formal heterogeneity testing (Cochran’s *Q* and *I*^2^; Supplementary Table S3) (32); standard ITS diagnostics including Shapiro–Wilk and Durbin–Watson tests (Supplementary Table S5); the two-breakpoint and weighted models described above (Supplementary Tables S6, S7); and models excluding fourth-year students, whose May testing date differs from the December date used for all other years (Supplementary Table S8).

#### 2.4.5 Data completeness

Of 300,535 measurement records eligible for the cardiorespiratory endurance ITS analysis (2008–2025), 2,970 (0.99%) were excluded because of missing or unparseable cardiorespiratory data. Excluded records comprised explicit non-participation entries (e.g., “not tested,” “abroad,” “exchange student”), zero or otherwise invalid run-time values, and run-time strings that could not be parsed by the automated extraction algorithm (*n* = 137, concentrated in 2011, with a mean value of 232 s consistent with the cohort mean, indicating no systematic bias). The final analytic sample comprised 297,565 valid cardiorespiratory records from 91,686 students. An additional 50,441 records from 2005 to 2007 were retained for the body-weight negative-control analysis.

Analyses were conducted in R 4.4.2 (2024-10-31 ucrt) using stats 4.4.2 for linear models, model comparison, and Shapiro–Wilk tests; sandwich 3.1.1 and lmtest 0.9.40 for Newey–West standard errors and Durbin–Watson testing; readxl 1.4.5 and openxlsx 4.2.7.1 for data import; dplyr 1.1.4 for data management; and ggplot2 3.5.2 for figures. False discovery rate correction used *p*.adjust in stats. Two-sided *p* < 0.05 was treated as the conventional threshold, with interpretive weight given to effect sizes and confidence intervals. Analysis scripts are available from the corresponding author on request.

## 3 Results

### 3.1 Sample characteristics

Across 2005–2025, 108,259 unique students contributed 350,976 measurements. For the cardiorespiratory analysis (2008–2025), the analytic sample comprised 297,565 valid records (150,286 from men, 147,279 from women) from 91,686 students, yielding 18 annual time points per sex. Annual sample sizes were stable across the series (women 7,273–8,809, coefficient of variation 0.051; men 7,430–9,362, CV 0.065). Characteristics by period appear in Table 1 and participant flow in Figure 1.

### 3.2 Cardiorespiratory endurance: pre-pandemic trend and change at 2020

Pre-pandemic trends in middle-distance run times were modest. In women, the 800-m time was decreasing (improving) before the pandemic (*β*_1_ = −0.90 s/year, 95% CI −1.45 to −0.36, *p* = 0.003); in men, the 1,000-m pre-pandemic trend was not statistically distinguishable from zero (*β*_1_ = −0.57 s/year, 95% CI −1.21 to +0.07, *p* = 0.077).

Relative to counterfactual projections, run times in 2020 were significantly slower in both sexes: +9.60 s in women (95% CI 2.58 to 16.62; *p* = 0.011; *q* = 0.019) and +13.02 s in men (95% CI 4.76 to 21.28; *p* = 0.005; *q* = 0.019). This estimate is, however, sensitive to which students contribute to the 2020 mean. When fourth-year students were excluded, the immediate change fell to +2.29 s in women (*p* = 0.435) and +5.27 s in men (*p* = 0.160), neither significant (Supplementary Table S8). Fourth-year students were tested in May 2020, weeks after strict quarantine ended, whereas all other years were tested that December after several months of partial resumption. The magnitude of the immediate change should therefore be interpreted as reflecting, in substantial part, the timing of testing relative to the most restrictive phase, and we do not treat it as a robust estimate of the average effect of the restriction period. Parameter estimates appear in Table 2 and fitted trajectories in Figure 2.

**Table 2:** Interrupted time series analysis of cardiorespiratory endurance, by sex.

Sex	Parameter	Estimate	95% CI	*p*	*q* (FDR)
Female (800 m)	*β*_1_ pre-pandemic trend (s/year)	−0.90	−1.45 to −0.36	0.003	—
	*β*_2_ immediate effect (s)	**+9.60**	2.58 to 16.62	**0.011**	0.019
	*β*_3_ trend change (s/year)	**+2.25**	0.60 to 3.90	**0.011**	0.019
	Model R^2^	0.794	—	—	—
Male (1,000 m)	*β_1_* pre-pandemic trend (s/year)	−0.57	−1.21 to 0.07	0.077	—
	*β_2_* immediate effect (s)	**+13.02**	4.76 to 21.28	**0.005**	0.019
	*β_3_* trend change (s/year)	−0.47	−2.41 to 1.47	0.609	0.609
	Model R^2^	0.553	—	—	—
Interaction	Sex × *β_3_* diff. (s/year)	**−2.72**	−5.15 to −0.29	**0.030**	0.037

Single-breakpoint interrupted time series (breakpoint at 2020) estimated by ordinary least squares. Positive values denote worsening (slower running times). *p* is two-sided; *q* is the Benjamini–Hochberg FDR-adjusted p-value across the five confirmatory cardiorespiratory tests. Bold estimates remain significant after FDR correction (*q* < 0.05). Residual diagnostics support OLS inference (Durbin–Watson = 2.41 female, 2.24 male; Shapiro–Wilk *p* = 0.88 and 0.57). The pre-specified body-weight ITS (negative control) is reported in Figure 4; the level-change and trend-change coefficients (β_2_, β_3_) were non-significant in both sexes.

**Figure 2:**
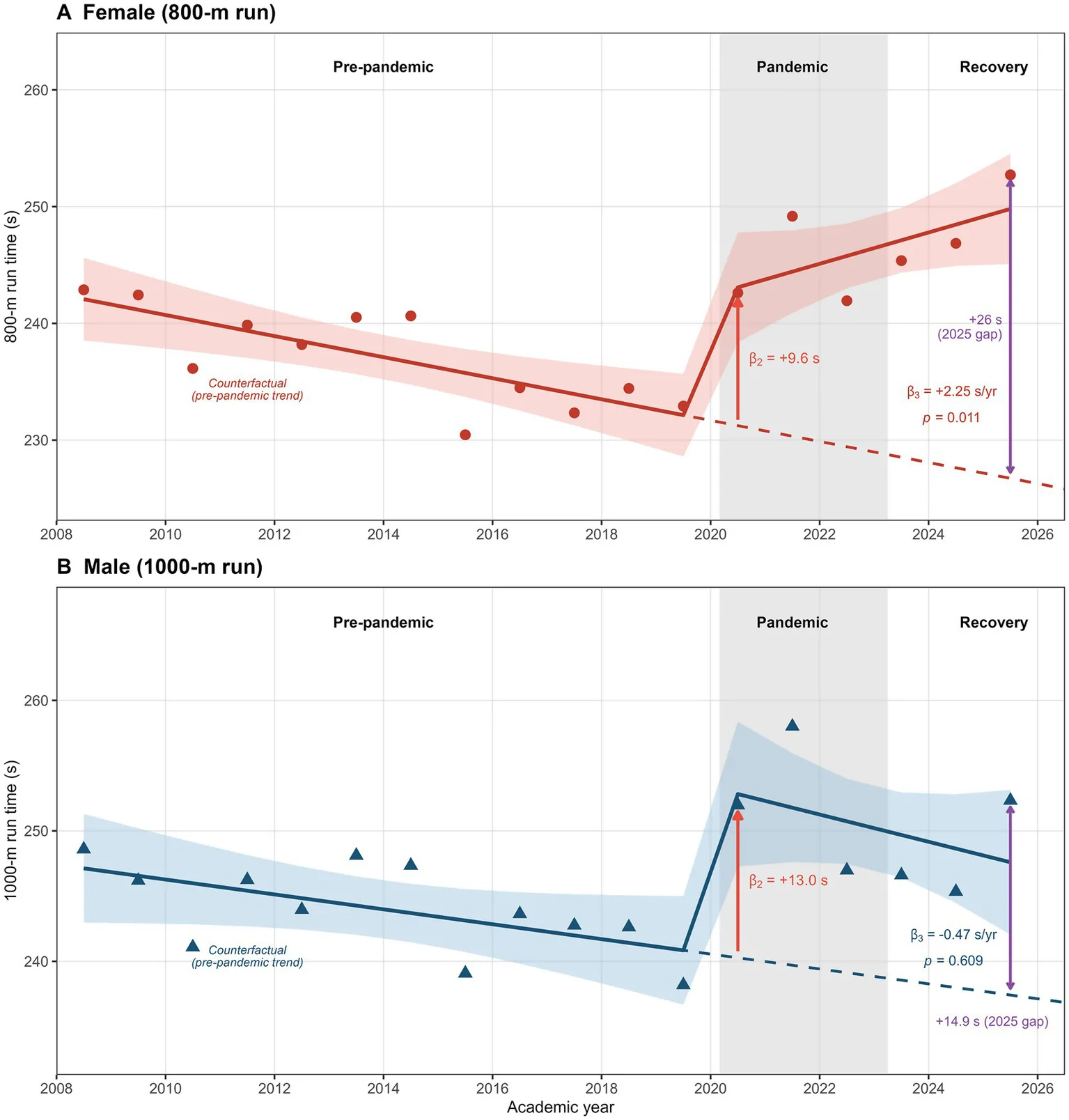
Interrupted time series analysis of cardiorespiratory endurance, by sex. **(A)** Female 800-m run time; **(B)** male 1,000-m run time. Points, observed grade-standardized annual means; solid lines, fitted values with 95% confidence bands; dashed lines, counterfactual projections from the pre-pandemic trend. Shading indicates the pandemic period. Annotations show the level change at 2020 (*β*_2_) and the change in annual trend thereafter (*β*_3_). Values are population-level annual means, not individual trajectories.

### 3.3 Sex differences in post-2020 trends

Post-2020 trends differed significantly between women and men (sex × time-after interaction *p* = 0.030; *q* = 0.037). In men, the post-2020 trend did not differ significantly from the pre-pandemic trend (*β*_3_ = −0.47 s/year, 95% CI −2.41 to +1.47; *p* = 0.609); the annual mean nonetheless remained approximately 14.9 s above the counterfactual projection in 2025. In women, the post-2020 trend was significantly steeper than the pre-pandemic trend (*β*_3_ = +2.25 s/year, 95% CI 0.60 to 3.90; *p* = 0.011; *q* = 0.019), so that the distance between observed annual means and the counterfactual projection widened to approximately 26.0 s by 2025. These are aggregate annual means from repeated cross-sections and describe population-level trends; they cannot be interpreted as the trajectories of individual students. Figure 2 displays the fitted series and Figure 3 quantifies the deviation from counterfactual by year.

**Figure 3:**
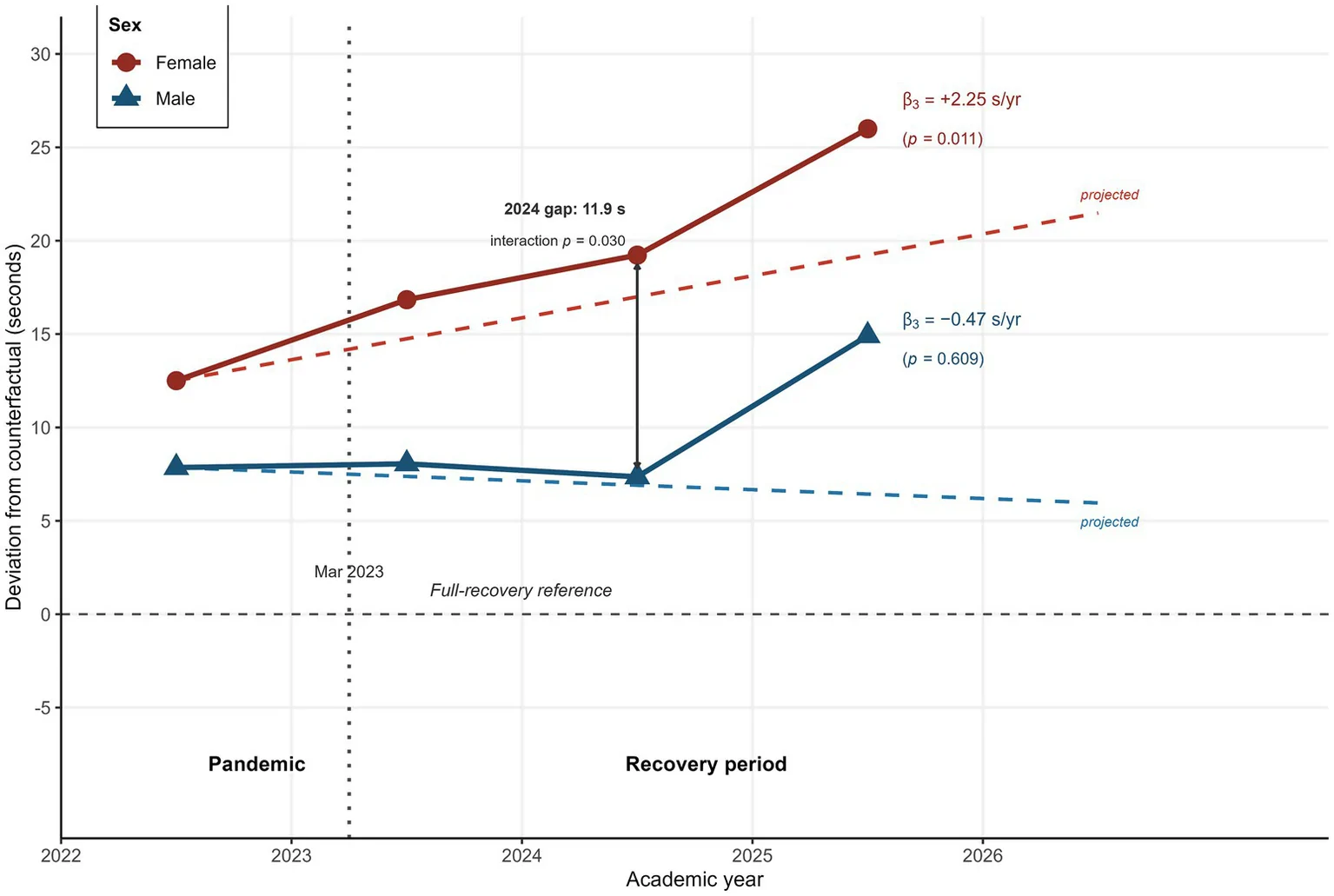
Deviation of observed annual means from counterfactual projections (seconds), by sex, 2022–2025. The difference between sexes in post-2020 trend was significant (*p* = 0.030).

Unlike the immediate change, this divergence was robust to the exclusion of fourth-year students. In that analysis the women’s trend change was +2.93 s/year (95% CI 1.50 to 4.37, *p* = 0.0006), the men’s +0.35 s/year (*p* = 0.682), and the interaction remained significant (−2.58, *p* = 0.022; Supplementary Table S8). It was also unchanged under alternative baseline windows (Supplementary Table S2), under sample-size weighting (interaction −2.69, *p* = 0.031; Supplementary Table S7), and after grade stratification (Supplementary Table S3).

Placing these figures on the national scoring scale gives a sense of their practical size. At 2.5 s per scoring point, the 26.0 s gap in women corresponds to approximately 10.4 points and the 14.9 s gap in men to approximately 6.0 points. In 2025 the observed female annual mean of 252.7 s scored approximately 68 points against a counterfactual projection of approximately 79 points, that is, a shift from just below the good threshold to the middle of the pass band; the corresponding male figures were approximately 67 against 73 points. Both sexes therefore remained within the passing range throughout, and we do not wish to overstate the change: no cohort mean fell below the pass threshold at any point in the series. What the scoring scale does show is that the difference between sexes is not only statistically detectable but also large enough to be visible in the administrative grading that students and institutions actually use.

### 3.4 Body weight as a negative control, and the secondary indicators

In contrast to the pandemic-specific disruption of cardiorespiratory endurance, body weight—analyzed within the identical ITS framework as a negative control—showed no pandemic-specific effect. Although body weight exhibited a strong long-term secular increase in both sexes (women: *β*_1_ = +0.21 kg/year; men: *β*_1_ = +0.50 kg/year; both *p* < 0.001), consistent with documented increases in adiposity among Chinese youth (11, 33, 34), neither the immediate effect nor the post-pandemic slope change reached significance (women: *β*_2_ = +0.39 kg, *p* = 0.31; *β*_3_ = +0.06 kg/year, *p* = 0.57; men: *β*_2_ = +0.30 kg, *p* = 0.70; *β*_3_ = +0.02 kg/year, *p* = 0.92; Figure 4). An indicator with a strong secular trend but no discontinuity at 2020, estimated in the same model that detected one for cardiorespiratory endurance, makes it less likely that the cardiorespiratory result is an artefact of the specification.

**Figure 4:**
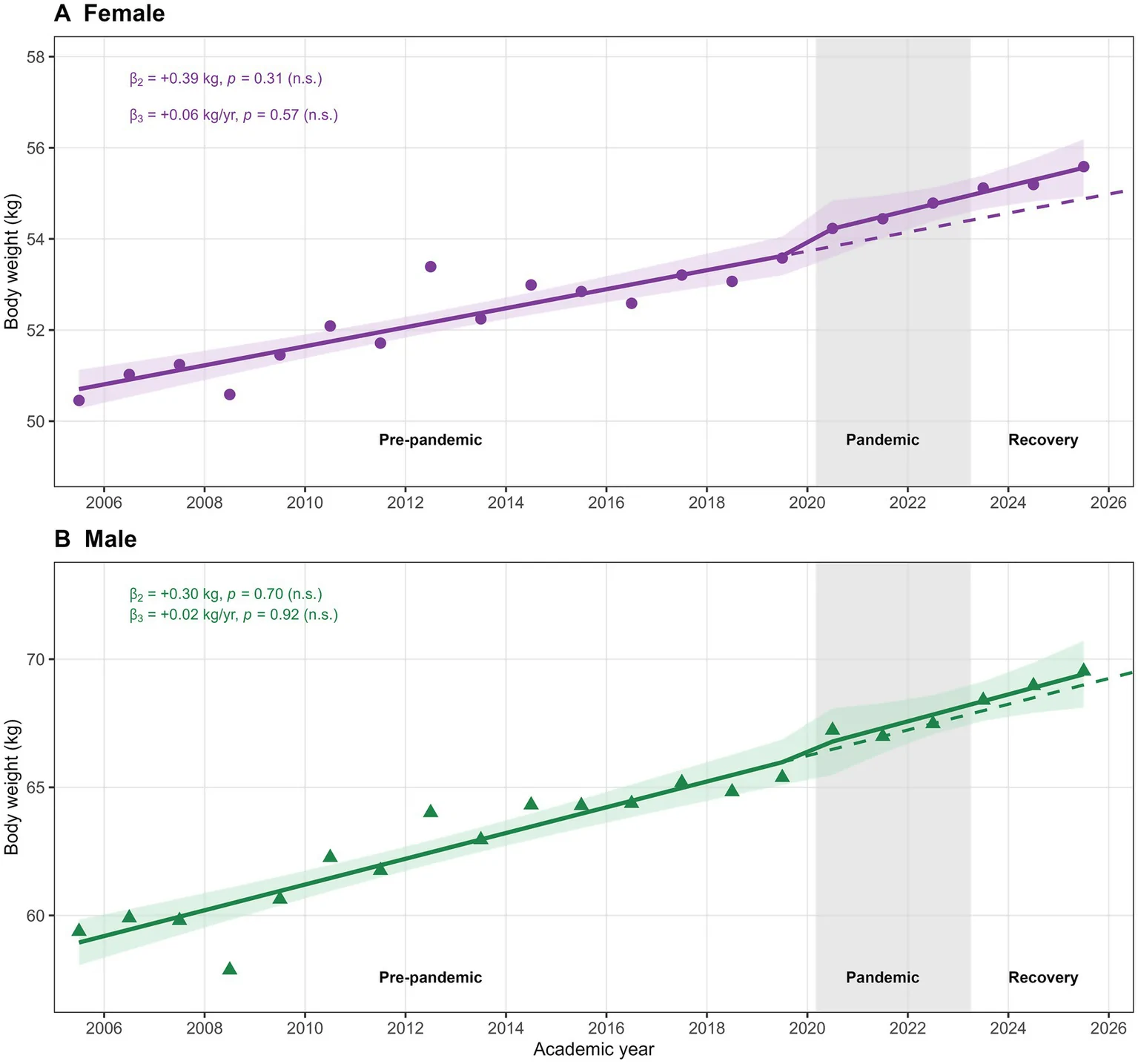
Interrupted time series analysis of body weight (negative control), by sex. **(A)** Female; **(B)** male. Conventions as in Figure 2. The absence of a pandemic-specific level or slope change contrasts with the cardiorespiratory result.

The four Tier 3 indicators (standing long jump, 50-m sprint, sit-and-reach, muscular endurance), assessed by pre-pandemic baseline comparison, showed small and directionally inconsistent changes (all |*d*| ≤ 0.46), with the largest effect being an improvement in flexibility rather than a deterioration (Table 3). We note that these indicators were not analysed by ITS, so this comparison is weaker than the one between cardiorespiratory endurance and body weight and is presented as exploratory.

**Table 3:** Baseline-referenced changes in secondary physical-fitness indicators (Tier 3) during the restriction and post-reopening periods, by sex.

Indicator	Sex	Baseline (2017–2019)	Restriction period Δ (%), *d*	Post-reopening Δ (%), *d*
Standing long jump (cm)	Women	174.55	−0.32 (−0.2%), −0.02	−2.97 (−1.7%), −0.18
	Men	231.30	−1.64 (−0.7%), −0.08	−4.97 (−2.2%), −0.24
50-m sprint (s)^a^	Women	8.94	+0.05 (+0.6%), +0.08	+0.09 (+1.0%), +0.15
	Men	7.29	+0.02 (+0.3%), +0.04	+0.02 (+0.2%), +0.04
Sit-and-reach (cm)	Women	18.91	+1.15 (+6.1%), +0.20	+2.62 (+13.9%), +0.46
	Men	15.51	+0.18 (+1.2%), +0.03	+1.76 (+11.4%), +0.26
Muscular endurance (reps)^b^	Women	38.28	+1.03 (+2.7%), +0.11	+1.62 (+4.2%), +0.16
	Men	6.84	−1.19 (−17.3%), −0.20	−1.07 (−15.6%), −0.17

Δ, deviation from grade-standardized baseline; *d*, Cohen’s *d* (pooled individual-level SD). Effects interpreted by magnitude (0.2 small, 0.5 medium, 0.8 large); this is an exploratory analysis.

a For 50-m sprint, positive values denote slower (poorer) times.

b Muscular endurance was assessed with one-minute sit-ups in women and pull-ups in men. The two tests are not equivalent and were never compared between sexes; all inference is within-sex.

### 3.5 Heterogeneity and model diagnostics

Grade-stratified models showed substantial heterogeneity in the immediate change (*β*_2_: *I*^2^ ≈ 89%; Cochran’s *Q p* < 0.001; Supplementary Table S3), driven by the fourth-year cohort for the reasons given in section 3.2. Heterogeneity in the trend change was moderate and not significant (*β*_3_: *I*^2^ ≈ 40%, *p* ≈ 0.18), indicating consistency of direction across grades.

Residual diagnostics were acceptable for the primary models (Durbin–Watson: women 2.41, men 2.24, both *p* > 0.39; Shapiro–Wilk: women *W* = 0.975, *p* = 0.881; men *W* = 0.958, *p* = 0.565; Supplementary Table S5). Model comparison did not favour a second breakpoint at 2023 in either sex [women ΔBIC = +3.30, *F*(2, 12) = 0.89, *p* = 0.437; men ΔBIC = +1.67, *F*(2, 12) = 1.54, *p* = 0.254], and neither 2023 parameter was significant (Supplementary Table S6). With only three annual observations after reopening, this comparison has limited power, and the result should be read as an inability to detect a discontinuity at 2023 rather than as evidence that none occurred.

## 4 Discussion

### 4.1 Principal findings

In 21 years of consistently measured data from 108,259 students, two findings stand out. First, cardiorespiratory endurance was the only indicator analysed within the ITS framework that showed a sustained change associated with the pandemic; body weight, analysed identically, continued along its long-term secular trajectory without interruption. Second, post-2020 trends in cardiorespiratory endurance differed by sex (interaction *p* = 0.030, *q* = 0.037): in men the trend did not differ significantly from the pre-pandemic trend, while in women it was significantly steeper, so that the gap from the counterfactual projection continued to widen through 2025. We are not aware of a previous report of this pattern.

Two qualifications belong here rather than at the end. First, the immediate 2020 level change is not robust to the exclusion of fourth-year students: when they are removed, the estimate falls from +9.60 s to +2.29 s in women and from +13.02 s to +5.27 s in men, and is no longer statistically significant in either sex (Supplementary Table S8). Its magnitude therefore depends substantially on when each cohort was tested relative to the strictest phase of restrictions, and we place little weight on its exact size. Second, the divergence in subsequent trends survived every sensitivity analysis we ran, including that exclusion, and it is on that finding that the paper’s contribution rests.

### 4.2 Comparison with existing literature

The immediate changes we observed are broadly consistent in direction with reported declines in Chinese student cohorts, with differences across studies attributable to sample composition, regional restriction intensity, and the timing of testing (5–10)—the last of which our own fourth-year analysis illustrates directly. Our contribution is threefold. The post-reopening window has not previously been examined at this scale, since most studies conclude during the restriction period or in early 2023 (5–12). The contrast between an indicator that changed and one that did not, estimated within a single framework on a single sample, has not been demonstrated in this literature. And the divergence of post-pandemic trends by sex appears to be new.

### 4.3 Why cardiorespiratory endurance and not the other indicators

The selective vulnerability of cardiorespiratory endurance has a well-established physiological basis. Aerobic capacity depends heavily on continuous training stimulus: classic detraining studies document declines in maximal oxygen uptake of 4–14% within a few weeks of training cessation, whereas strength and neuromuscular adaptations decline far more slowly over comparable intervals (13–15). A 2020 lockdown of several months’ duration would therefore be expected to produce large, persistent effects on aerobic capacity but minimal effects on strength and flexibility. A secondary environmental contributor is space dependence: middle-distance running requires open outdoor venues that became inaccessible during campus closures, whereas basic strength and flexibility exercises can be performed in confined indoor settings.

The empirical pattern in our data is consistent with both explanations, but we want to be careful about how far it can be pushed. Body weight and cardiorespiratory endurance were analysed identically, so the contrast between them is meaningful. The four secondary indicators were not: they were assessed by baseline comparison rather than ITS, because their series were too short, and a difference in analytical method is a poor basis for a claim about differential vulnerability. We therefore conclude only that cardiorespiratory endurance changed and body weight did not, and treat the secondary indicators as broadly reassuring but not as evidence of relative robustness.

The body-weight negative control merits brief emphasis. Body weight rose steadily across the entire observation window (secular trends of +0.21 and +0.50 kg/year in women and men, respectively), in line with well- documented secular changes in body size and physical fitness among young people in China and elsewhere (33–36); yet it showed no abrupt level or slope change attributable to the pandemic. This dissociation—an indicator with a strong secular trend but no pandemic-specific signal, embedded in the same model that detected a clear cardiorespiratory effect—makes it less likely that our primary result is generated by the model specification. It does not, however, exclude all sources of bias: any confounder acting on running performance but not on body weight would survive this check, and that is precisely the class of confounder one would worry about here.

### 4.4 Possible explanations for the sex difference

We measured no behavioral, psychological, or physiological mediators, so what follows are hypotheses suggested by the pattern rather than findings. We set them out with the data that would be needed to test each.

The first is a difference in how physical activity resumed. Male students in Chinese universities participate more frequently in competitive and team-based sport, which restarted quickly once restrictions ended, whereas women’s activity is comparatively concentrated in individual or class-based formats that may have resumed more gradually (37). If men returned to vigorous aerobic activity sooner, a less steep post-2020 trend at the population level would follow from the same detraining physiology described above. Testing this requires activity logs or accelerometry alongside the fitness testing.

The second is the psychosocial aftermath. A disproportionate burden of pandemic-related anxiety, depression, and disrupted health behavior among young women is well documented (18–20), and psychological distress is associated with lower exercise motivation and activity (12). If these effects were larger or more persistent in women, they could delay the sustained training needed to reverse aerobic decline. Testing this requires repeated mental health measurement linked to individual fitness records.

The third concerns baseline physiology and body composition. Women have lower absolute baseline cardiorespiratory fitness, so an equivalent interruption may represent a larger relative decrement and be harder to reverse (16, 17); concurrent changes in body composition could further impede weight-bearing aerobic performance. Testing this requires body composition assessment rather than body weight alone, which our data do not contain.

What these accounts share is that they would locate the difference in modifiable behavioral and psychosocial circumstances rather than in fixed biology. If any of them is correct, the divergence would be amenable to intervention. We cannot distinguish among them with the data available, we cannot exclude explanations we have not considered, and none of these accounts should be read as a finding of this study.

### 4.5 Seasonality and the timing of testing

First- to third-year students were tested each December and fourth-year students each May. These months differ in temperature, daylight, and position within the academic and training year, all of which influence middle-distance running performance, and this asymmetry is a plausible contributor to the large between-grade heterogeneity we observed in the immediate change. Because the testing month for each grade was the same in every study year, seasonality is unlikely on its own to generate a discontinuity at 2020 in a grade-standardised series; we would nonetheless not treat it as excluded. Seasonal conditions interact with the pandemic timeline rather than running parallel to it, since the degree of restriction differed between the May and December testing windows of 2020, and we have no measurements of temperature, track condition, or training exposure with which to separate these influences. Seasonality therefore remains a source of uncertainty in the immediate estimate as well as a likely contributor to between-grade heterogeneity. The trend parameters, which compare slopes across years within an unchanged seasonal structure, are less exposed to this problem than the level change, but they are not immune to it.

### 4.6 Implications

If the divergence we observe is real and not peculiar to this institution, it has a straightforward programmatic implication: aerobic fitness in women should be treated as a distinct priority rather than as one element of a generic post-pandemic fitness package, and sex-tailored approaches—women-centred aerobic programming, peer-led running groups, attention to motivational and scheduling barriers that differ by sex—warrant evaluation. For emergency preparedness more broadly, our results are consistent with the view that continuity of aerobic activity requires explicit planning, because unlike strength work it cannot be maintained adequately in confined indoor space.

### 4.7 Strengths and limitations

The principal strengths of this study are its 21-year observation window, its large analytic sample, and its single-site measurement consistency. The extended pre-pandemic baseline enabled formal counterfactual modeling rather than simple before-and-after comparison, and the use of one testing team with stable equipment and protocols across two decades minimized the measurement heterogeneity that typically obscures temporal trends in multi-site fitness studies. The pre-specified hypothesis framework, the embedded negative control, the formal model-selection and sensitivity analyses, and false discovery rate correction together provide a level of analytical rigor uncommon in this literature.

The limitations are substantial and shape what can be concluded. This is one comprehensive public university in one province, with its own admissions profile and physical education curriculum; restriction intensity and reopening timing varied considerably across China and internationally, and the magnitudes reported here should not be assumed to transfer. The design is a repeated cross-section of annual means, so the results describe population-level trends and cannot characterise individual trajectories or test the mechanisms proposed above. Although the individual-level sample is large, the ITS models rest on 18 annual observations per sex, of which only three follow full reopening (38); this limits both the precision of the post-reopening estimates and the power of the two-breakpoint comparison. The immediate 2020 estimate is sensitive to the testing-time asymmetry between grades, as discussed. The cross-indicator comparison is not methodologically uniform. And apart from the middle-distance run, for which the national standard provides scoring thresholds, we lacked contemporaneous normative data against which to judge the functional significance of the observed changes, so interpretation of the four secondary indicators is confined to relative change within the cohort.

### 4.8 Future directions

Four priorities follow directly from these limitations. Replication across multiple sites that differed in restriction intensity and reopening timing would establish whether the divergent trend pattern generalises or is local. Individual-level cohorts linking annual fitness testing to repeated measurement of physical activity and mental health are the only design capable of separating the candidate mechanisms and of distinguishing population trends from individual change (39). Continued surveillance beyond 2025 is needed, since three post-reopening observations cannot establish whether the trend in women stabilises, reverses, or persists. And intervention studies testing whether sex-tailored aerobic programming alters the trajectory would convert an observational signal into actionable evidence.

## 5 Conclusion

Among 108,259 Chinese university students measured over 21 years, cardiorespiratory endurance was the only fitness component analysed by interrupted time series that showed a sustained change associated with the COVID-19 pandemic; body weight, analysed in the same framework as a negative control, remained on its long-term secular trajectory. This contrast makes a purely methodological explanation less likely, but it does not exclude confounding, and in particular it cannot rule out influences acting on running performance and not on body weight. The four remaining indicators were assessed by a different and weaker method, so no claim of relative vulnerability is made for them. Post-2020 trends in cardiorespiratory endurance differed by sex: in men the trend did not differ significantly from the pre-pandemic trend, whereas in women it was significantly steeper, with the gap from the counterfactual projection continuing to widen through 2025. These are population-level trends rather than individual trajectories, they come from a single institution, and the magnitude of the immediate 2020 change was not robust to the exclusion of fourth-year students. Within those limits, they suggest that aerobic fitness in young women warrants specific attention in post-pandemic health programming, and that continuity of aerobic activity should be considered explicitly when activity-restricting measures are planned.

## Data Availability

The aggregate datasets analyzed in this study are available from the corresponding author upon reasonable request. Individual-level data cannot be made publicly available owing to privacy restrictions on student educational records under Chinese data protection regulations and the conditions of the institutional ethics approval.
