# Supplementary material for: Sex-divergent trends in cardiorespiratory endurance after the COVID-19 pandemic: a 21-year interrupted time series analysis of Chinese university students
Source: Front Public Health. 2026 Oct 5;14:1910319. doi: 10.3389/fpubh.2026.1910319 (PMC13677492; doi:10.3389/fpubh.2026.1910319)
Supplement: Supplementary file 1 [file Supplementary_file_1.DOCX]

**STROBE Statement**

**Checklist of items that should be included in reports of cross-sectional studies**

*Manuscript 1910319. Sex-divergent trends in cardiorespiratory endurance after the COVID-19 pandemic: a 21-year interrupted time series analysis of Chinese university students.*

*Section and item numbers refer to the revised manuscript submitted with this response.*

| **Item** | **STROBE recommendation** | **Location in the revised manuscript** |
| --- | --- | --- |
| 1a | Indicate the study design with a commonly used term in the title or the abstract | Title (“interrupted time series analysis”); Abstract, sentence 3 |
| 1b | Provide in the abstract an informative and balanced summary of what was done and what was found | Abstract (single paragraph; design, sample, methods, results, and limits of interpretation) |
| 2 | Explain the scientific background and rationale for the investigation being reported | Introduction, paragraphs 1–6 |
| 3 | State specific objectives, including any prespecified hypotheses | Introduction, final paragraph (aims) |
| 4 | Present key elements of study design early in the paper | Methods 2.1 (repeated cross-sectional design with interrupted time series analysis) |
| 5 | Describe the setting, locations, and relevant dates, including periods of recruitment, exposure, follow-up, and data collection | Methods 2.1–2.2 (one comprehensive public university, Zhejiang Province; 2005–2025; three pre-defined periods) |
| 6 | Give the eligibility criteria, and the sources and methods of selection of participants | Methods 2.2 (explicit inclusion and exclusion criteria; complete administrative census) |
| 7 | Clearly define all outcomes, exposures, predictors, potential confounders, and effect modifiers | Methods 2.3 (six indicators, components assessed, tier classification); Methods 2.4.2 (model parameters) |
| 8 | For each variable of interest, give sources of data and details of methods of assessment | Methods 2.3 (National Student Physical Fitness Standard protocol; test order, warm-up, rest intervals, venue, time of day, testing conditions) |
| 9 | Describe any efforts to address potential sources of bias | Methods 2.4.1 (grade standardisation); 2.4.4 (five sensitivity analyses); 2.4.5 (completeness and exclusions) |
| 10 | Explain how the study size was arrived at | Methods 2.2 (complete census; no a priori sample size calculation; power governed by number of annual time points) |
| 11 | Explain how quantitative variables were handled in the analyses | Methods 2.4.1 (annual grade-standardised means as unit of analysis); 2.4.2 (ITS specification) |
| 12a | Describe all statistical methods, including those used to control for confounding | Methods 2.4.2–2.4.3 (OLS-ITS; Newey–West check; sex × trend interaction; Benjamini–Hochberg correction) |
| 12b | Describe any methods used to examine subgroups and interactions | Methods 2.4.3 (sex × time-after interaction); 2.4.4 (grade-stratified models) |
| 12c | Explain how missing data were addressed | Methods 2.4.5 (2,970 records excluded, 0.99%; nature of exclusions described) |
| 12d | If applicable, describe analytical methods taking account of sampling strategy | Not applicable — complete census, no sampling (Methods 2.2) |
| 12e | Describe any sensitivity analyses | Methods 2.4.4 (baseline windows; grade stratification; diagnostics; two-breakpoint model; sample-size weighting; exclusion of fourth-year students) |
| 13a | Report numbers of individuals at each stage of the study | Results 3.1; Figure 1 |
| 13b | Give reasons for non-participation at each stage | Methods 2.4.5; Figure 1 |
| 13c | Consider use of a flow diagram | Figure 1 |
| 14a | Give characteristics of study participants and information on exposures and potential confounders | Table 1; Results 3.1 |
| 14b | Indicate number of participants with missing data for each variable of interest | Methods 2.4.5; Figure 1 |
| 15 | Report numbers of outcome events or summary measures | Results 3.2–3.4; Tables 2 and 3 |
| 16a | Give unadjusted estimates and, if applicable, confounder-adjusted estimates and their precision | Table 2 (estimates with 95% confidence intervals); Results 3.2–3.3 |
| 16b | Report category boundaries when continuous variables were categorised | Not applicable — no continuous variable was categorised |
| 16c | If relevant, consider translating estimates of relative risk into absolute risk | Results 3.3 (effects expressed in seconds and translated onto the national fitness scoring scale) |
| 17 | Report other analyses done | Results 3.4 (body-weight negative control; Tier 3 baseline comparison); Results 3.5 (heterogeneity, diagnostics, two-breakpoint comparison); Supplementary Tables S1–S8 |
| 18 | Summarise key results with reference to study objectives | Discussion 4.1 |
| 19 | Discuss limitations of the study, taking into account sources of potential bias or imprecision | Discussion 4.7; also 4.3 (limits of the negative control) and 4.5 (seasonality and testing time) |
| 20 | Give a cautious overall interpretation of results considering objectives, limitations, multiplicity of analyses, results from similar studies, and other relevant evidence | Discussion 4.1–4.6 |
| 21 | Discuss the generalisability (external validity) of the study results | Discussion 4.7 (single-site design; variation in restriction intensity and reopening timing) |
| 22 | Give the source of funding and the role of the funders | Declarations — Funding |

*All 22 STROBE items for cross-sectional studies are addressed in the revised manuscript. Items 12d and 16b are not applicable to this design and are marked as such.*

*Reference: von Elm E, Altman DG, Egger M, Pocock SJ, Gøtzsche PC, Vandenbroucke JP. The Strengthening the Reporting of Observational Studies in Epidemiology (STROBE) statement: guidelines for reporting observational studies. Lancet. (2007) 370:1453–7.*
