# Supplementary material for: Sex-divergent trends in cardiorespiratory endurance after the COVID-19 pandemic: a 21-year interrupted time series analysis of Chinese university students
Source: Front Public Health. 2026 Oct 5;14:1910319. doi: 10.3389/fpubh.2026.1910319 (PMC13677492; doi:10.3389/fpubh.2026.1910319)
Supplement: Supplementary file 2 [file Supplementary_file_2.DOCX]

**Supplementary Figure S1. Study design framework. Panel A, the COVID-19 timeline and the three pre-defined study periods (pre-pandemic baseline, 2005–February 2020; pandemic, February 2020–February 2023; post-reopening, March 2023–2025). Panel B, the indicator-tiering strategy based on data availability and interrupted time series (ITS) diagnostic suitability: Tier 1 (body weight) and Tier 2 (cardiorespiratory endurance, the primary outcome) were analysed by ITS, whereas Tier 3 indicators were assessed by pre-pandemic baseline comparison. Panel C, the three-layer analytical framework, comprising grade standardization as the methodological foundation, primary effect analysis (Layer 1), pre-specified heterogeneity exploration (Layer 2), and sensitivity and robustness checks (Layer 3). ITS, interrupted time series; H1–H3, study hypotheses 1–3; HAC, heteroskedasticity- and autocorrelation-consistent; FDR, false discovery rate.**

**Table S1. Comparison of single- versus two-breakpoint interrupted time series models for cardiorespiratory endurance**

| **Sex** | **AIC (single)** | **AIC (double)** | **ΔAIC** | **BIC (single)** | **BIC (double)** | **ΔBIC** | **F (df)** | **p** |
| --- | --- | --- | --- | --- | --- | --- | --- | --- |
| Female (800-m run) | 96.55 | 98.06 | +1.52 | 101.00 | 104.30 | +3.30 | 0.89 (2,12) | 0.437 |
| Male (1000-m run) | 102.40 | 102.29 | −0.11 | 106.85 | 108.52 | +1.67 | 1.54 (2,12) | 0.254 |

*Note.* ΔAIC and ΔBIC = double-breakpoint minus single-breakpoint; positive values favour the single-breakpoint model. The F-statistic tests whether the two additional parameters (level change and slope change at 2023) jointly improve fit. Neither sex showed improved fit with a second breakpoint (all p > 0.25).

**Table S2. Sensitivity of ITS estimates to baseline window selection**

| **window** | **Sex** | **β₂ (s)** | **p(β₂)** | **β₃ (s/yr)** | **p(β₃)** | **Baseline mean (s)** | **Post-reopening mean (s)** | **Difference (s)** | **Difference (%)** |
| --- | --- | --- | --- | --- | --- | --- | --- | --- | --- |
| 2015–2019 | Female | 9.60 | 0.011 | 2.25 | 0.011 | 232.92 | 248.32 | +15.39 | +6.6% |
|  | Male | 13.02 | 0.005 | −0.47 | 0.609 | 241.26 | 248.10 | +6.84 | +2.8% |
| 2016–2019 | Female | 9.60 | 0.011 | 2.25 | 0.011 | 233.54 | 248.32 | +14.77 | +6.3% |
|  | Male | 13.02 | 0.005 | −0.47 | 0.609 | 241.80 | 248.10 | +6.29 | +2.6% |
| 2017–2019 (primary) | Female | 9.60 | 0.011 | 2.25 | 0.011 | 233.23 | 248.32 | +15.09 | +6.5% |
|  | Male | 13.02 | 0.005 | −0.47 | 0.609 | 241.19 | 248.10 | +6.91 | +2.9% |

*Note. ITS models were fitted to the full 2008–2025 annual series in all specifications; the baseline window affects only the descriptive comparison of post-reopening means. β₂ = immediate level change at 2020; β₃ = post-2020 trend change. ITS coefficients were identical across all three windows; the maximum difference in baseline means was <0.3% of the baseline value. Post-reopening mean = 2023–2025 average. The 2017–2019 window was pre-specified as the primary analysis.*

**Table S3. Grade-stratified interrupted time series estimates and heterogeneity statistics**

| **Sex** | **Grade** | **β₂ (s)** | **SE(β₂)** | **p(β₂)** | **β₃ (s/yr)** | **SE(β₃)** | **p(β₃)** |
| --- | --- | --- | --- | --- | --- | --- | --- |
| Female | Grade 1 | 1.11 | 3.02 | 0.720 | 2.25 | 0.81 | 0.014 |
|  | Grade 2 | 5.89 | 2.55 | 0.037 | 3.42 | 0.68 | <0.001 |
|  | Grade 3 | 8.88 | 3.50 | 0.024 | 3.15 | 0.93 | 0.005 |
|  | Grade 4 | 32.57 | 5.37 | <0.001 | 0.09 | 1.43 | 0.948 |
|  | Pooled (precision-weighted) | 7.59 | — | — | 2.72 | — | — |
|  | Heterogeneity: Q (df = 3) | 26.84 | — | <0.001 | 4.97 | — | 0.174 |
|  | I² | 88.8% | — | — | 39.7% | — | — |
| Male | Grade 1 | 0.39 | 3.23 | 0.905 | −0.33 | 0.86 | 0.705 |
|  | Grade 2 | 7.99 | 3.47 | 0.037 | 0.49 | 0.92 | 0.602 |
|  | Grade 3 | 8.67 | 4.20 | 0.058 | 0.92 | 1.12 | 0.425 |
|  | Grade 4 | 34.22 | 5.82 | <0.001 | −3.05 | 1.55 | 0.070 |
|  | Pooled (precision-weighted) | 8.32 | — | — | −0.11 | — | — |
|  | Heterogeneity: Q (df = 3) | 25.85 | — | <0.001 | 4.92 | — | 0.178 |
|  | I² | 88.4% | — | — | 39.1% | — | — |

*Note. Each grade-level model was fitted to the full 2008–2025 annual series (18 observations per grade per sex). β₂ = immediate level change at 2020; β₃ = post-2020 trend change. Pooled estimates are precision-weighted means (w = 1/SE²). Substantial heterogeneity in β₂ (I² ≈ 89%, Q p < 0.001) was driven primarily by Grade 4, whose markedly higher immediate disruption reflects elevated pre-graduation test disengagement compounded by pandemic conditions; this grade-level variation is the primary rationale for grade standardisation in the main analysis. Heterogeneity in β₃ was moderate and non-significant (I² ≈ 40%, p ≈ 0.18), supporting directional consistency of post-2020 trends across grades.*

**Table S4. Benjamini–Hochberg false-discovery-rate correction for the pre-specified confirmatory hypothesis family**

| **Test** | **Estimate** | **Raw p** | **Adjusted q** |
| --- | --- | --- | --- |
| Cardiorespiratory endurance — confirmatory family (k = 5) |  |  |  |
| Female 800-m: immediate level change, β₂ (s) | +9.60 | 0.011 | 0.019 |
| Female 800-m: post-2020 trend change, β₃ (s/yr) | +2.25 | 0.011 | 0.019 |
| Male 1000-m: immediate level change, β₂ (s) | +13.02 | 0.005 | 0.019 |
| Male 1000-m: post-2020 trend change, β₃ (s/yr) | −0.47 | 0.609 | 0.609 |
| Sex × post-2020 trend change interaction (s/yr) | −2.72 | 0.030 | 0.037 |
| Body weight — negative control (not FDR-adjusted) |  |  |  |
| Female: immediate level change, β₂ (kg) | +0.39 | 0.309 | — |
| Female: post-2020 trend change, β₃ (kg/yr) | +0.06 | 0.566 | — |
| Male: immediate level change, β₂ (kg) | +0.30 | 0.699 | — |
| Male: post-2020 trend change, β₃ (kg/yr) | +0.02 | 0.916 | — |

*Note. Benjamini–Hochberg correction was applied within the pre-specified confirmatory family of five cardiorespiratory tests (β₂ and β₃ for each sex, plus the sex × post-2020 trend change interaction). Body-weight ITS parameters were pre-specified as a negative control and are reported separately without FDR adjustment, as a non-significant falsification check is not a member of the discovery family. All four primary confirmatory tests remained significant after correction (q < 0.05).*

**Table S5. OLS model diagnostics for interrupted time series models**

| **Outcome** | **Sex** | **n** | **R²** | **Adj. R²** | **F (df)** | **p(F)** | **DW** | **p(DW)** | **SW W** | **p(SW)** | **Residual SE** |
| --- | --- | --- | --- | --- | --- | --- | --- | --- | --- | --- | --- |
| Cardiorespiratory endurance | Female (800-m) | 18 | 0.794 | 0.750 | 17.96 (3,14) | <0.001 | 2.412 | 0.552 | 0.975 | 0.881 | 3.037 s |
|  | Male (1000-m) | 18 | 0.552 | 0.457 | 5.76 (3,14) | 0.009 | 2.242 | 0.398 | 0.958 | 0.565 | 3.573 s |
| Body weight (negative control) | Female | 20 | 0.931 | 0.918 | 71.62 (3,16) | <0.001 | 2.435 | 0.622 | 0.867 | 0.010 | 0.421 kg |
|  | Male | 20 | 0.936 | 0.924 | 78.00 (3,16) | <0.001 | 1.859 | 0.150 | 0.870 | 0.012 | 0.878 kg |

*Note.* Durbin–Watson (DW) p-values are one-sided tests for positive autocorrelation. Shapiro–Wilk (SW) tests normality of OLS residuals. Cardiorespiratory endurance models passed all diagnostics (DW p > 0.39, SW p > 0.56), supporting OLS inference. Body-weight negative-control models showed mild non-normality (SW p ≈ 0.01) but no autocorrelation (DW p > 0.15); given their role as a falsification check and the robustness of the non-significant findings, this mild violation does not affect interpretation.

*Additional sensitivity analyses (Tables S6–S8) were added in response to peer review.*

**Table S6. Full parameter estimates from two-breakpoint interrupted time series models (breakpoints at 2020 and 2023), by sex**

| **Sex / parameter** | **Estimate** | **95% CI** | **p** |
| --- | --- | --- | --- |
| **Female (800-m run)** |  |  |  |
| β₁ pre-pandemic trend (s/year) | −0.90 | −1.46 to −0.35 | 0.004 |
| β₂ level change at 2020 (s) | +13.11 | 2.30 to 23.92 | 0.022 |
| β₃ trend change after 2020 (s/year) | +0.57 | −4.18 to 5.32 | 0.800 |
| Level change at 2023 (s) | −3.27 | −15.14 to 8.60 | 0.560 |
| Trend change after 2023 (s/year) | +4.01 | −2.66 to 10.68 | 0.215 |
| **Male (1000-m run)** |  |  |  |
| β₁ pre-pandemic trend (s/year) | −0.57 | −1.20 to 0.06 | 0.071 |
| β₂ level change at 2020 (s) | +16.47 | 4.31 to 28.63 | 0.012 |
| β₃ trend change after 2020 (s/year) | −1.92 | −7.26 to 3.42 | 0.448 |
| Level change at 2023 (s) | −7.45 | −20.80 to 5.90 | 0.247 |
| Trend change after 2023 (s/year) | +5.35 | −2.15 to 12.86 | 0.146 |

*Note. This table reports the full coefficient set from the two-breakpoint specification whose fit was compared with the single-breakpoint model in Table S1. Neither parameter associated with the 2023 breakpoint reached significance in either sex, and information criteria did not favour the larger model (ΔBIC = +3.30 in women, +1.67 in men; nested F-tests p = 0.437 and p = 0.254; Table S1). With only three annual observations after full reopening (2023–2025), this comparison has limited statistical power; the absence of a detectable discontinuity at 2023 should not be read as positive evidence that no change occurred. Positive values denote worsening (slower run times). CI, confidence interval.*

**Table S7. Sample-size-weighted (WLS) interrupted time series estimates compared with unweighted (OLS) estimates**

| **Sex / parameter** | **OLS estimate** | **WLS estimate** | **WLS 95% CI** | **WLS p** |
| --- | --- | --- | --- | --- |
| Female β₁ (s/year) | −0.903 | −0.904 | −1.46 to −0.35 | 0.004 |
| Female β₂ (s) | +9.60 | +9.66 | 2.66 to 16.66 | 0.010 |
| Female β₃ (s/year) | +2.246 | +2.246 | 0.60 to 3.90 | 0.011 |
| Male β₁ (s/year) | −0.571 | −0.571 | −1.21 to 0.07 | 0.076 |
| Male β₂ (s) | +13.02 | +12.93 | 4.61 to 21.25 | 0.005 |
| Male β₃ (s/year) | −0.474 | −0.446 | −2.36 to 1.47 | 0.626 |
| Sex × β₃ interaction | −2.719 (p = 0.030) | −2.692 | — | 0.031 |

*Note. Each annual grade-standardised mean was weighted by its underlying sample size. Annual sample sizes were highly stable across the series (women 7,273–8,809, coefficient of variation 0.051; men 7,430–9,362, CV 0.065), and weighted estimates were therefore nearly identical to unweighted ones. Every substantive inference, including the sex × trend interaction, was unchanged.*

**Table S8. Interrupted time series estimates excluding fourth-year students (first- to third-year students only)**

| **Sex / parameter** | **Estimate** | **95% CI** | **p** | **R²** |
| --- | --- | --- | --- | --- |
| Female β₁ (s/year) | −0.66 | −1.14 to −0.19 | 0.009 | 0.770 |
| Female β₂ (s) | +2.29 | −3.81 to 8.39 | 0.435 | — |
| Female β₃ (s/year) | +2.93 | 1.50 to 4.37 | 0.0006 | — |
| Male β₁ (s/year) | −0.30 | −0.89 to 0.29 | 0.291 | 0.316 |
| Male β₂ (s) | +5.27 | −2.34 to 12.88 | 0.160 | — |
| Male β₃ (s/year) | +0.35 | −1.44 to 2.14 | 0.682 | — |
| Sex × β₃ interaction | −2.58 | — | 0.022 | — |

*Note. Analysis restricted to first- to third-year students (225,463 records), with grade weights renormalised to 0.3412 / 0.3363 / 0.3225. Fourth-year students were tested each May and all other years each December; excluding them removes the May 2020 observations taken only weeks after strict quarantine ended. The immediate level change at 2020 was attenuated and no longer significant in either sex, indicating that its magnitude in the primary model depends substantially on fourth-year students and the associated testing-time asymmetry. The post-2020 trend divergence, which is the principal finding, was unchanged or strengthened, and the sex × trend interaction remained significant.*
